# Trends and correlates of sexually transmitted infections among sexually active Ugandan female youths: evidence from three demographic and health surveys, 2006–2016

**DOI:** 10.1186/s12879-020-05732-x

**Published:** 2021-01-13

**Authors:** Veronicah Masanja, Solomon Tsebeni Wafula, Tonny Ssekamatte, John Bosco Isunju, Richard K. Mugambe, Guido Van Hal

**Affiliations:** 1 Department of Epidemiology and social medicine, Faculty of Medicine and Health Sciences, University of Antwerp, Antwerp, Belgium; 2 Department of Disease Control and Environmental Health, Makerere University, Kampala, Uganda

**Keywords:** Sexually transmitted infections, Female youths, Trends, Correlates, Uganda

## Abstract

**Background:**

Female adolescents and young women have the highest risk of curable sexually transmitted infections (STIs) globally. Data on the prevalence of STIs among young women in Uganda are limited. In this study, we investigated the time trends and correlates of STIs among adolescent girls and young women (15–24 years) in Uganda.

**Methods:**

We estimated the percentage of women 15–24 years from three recent consecutive Uganda Demographic and Health Surveys (2006, 2011, and 2016), who reported suffering from genital sores, and or genital discharge or any other varginal complaints acquired after sexual intercourse within 12 months of the studies and examined the changes over time. A pooled multivariable logistic regression was used to examine the correlates of reporting an STI in the last 12 months preceding the study. Svyset command in Stata was used to cater for the survey sample design.

**Results:**

The pooled self-reported STI prevalence was 26.0%. Among these young women, 22.0, 36.3, and 23.1% reported a sexually transmitted infection in 2006, 2011, and 2016 respectively. Between 2006 and 2011, there was evidence of change (+ 14.3%, *p* < 0.001) in STI prevalence before a significant reduction (− 12.0%, *p*< 0.001) in 2016. Youths aged 20–24 years reported a higher STI prevalence (27.3%) compared to young participants (23.6%). Correlates of reporting an STI among rural and urban young women were: having multiple total lifetime partners (adjusted odds ratio (aOR 1.6, 95% CI 1.4–1.6), being sexually active in the last 4 weeks (aOR 1.3, 95% CI 1.1–1.6), and being affiliated to Muslim faith (aOR 1.3, 95% CI 1.1–1.6) or other religions (aOR 1.8, 95% CI 1.1–2.9) as compared to Christian were more likely to report an STI. Living in Northern Uganda compared to living in Kampala city was found protective against STIs (aOR 0.5, 95% CI 0.3–0.7).

**Conclusion:**

The prevalence of STIs was high among female youths, 15–24 years. This highlights the need for a comprehensive STIs screening, surveillance, and treatment programme to potentially reduce the burden of STIs in the country.

## Background

Globally, the burden of sexually transmitted infections (STIs) remains a high public health concern. It is estimated that more than 1 million curable STIs are acquired every day worldwide, and 376 million new cases occur each year [1]. The burden of STIs is disproportionately higher in low and middle-income (LMIC) settings where an estimated 75–85% of new cases occur globally [2, 3]. If left untreated, STIs can result in adverse sexual, reproductive, and maternal-child health consequences including infertility, increased HIV risk, pelvic inflammatory diseases, ectopic pregnancies, and perinatal transmissions among others [4–6].

Although STIs affect all age groups, adolescents and young people aged 15–24 years are particularly more vulnerable [7, 8]. A study across three primary African regions (Southern Africa, Southern/Eastern Africa community based and East Africa high risk) revealed that all STIs except herpes simplex virus 2 (HSV 2) were more prevalent among young women, 15–24 years compared to those aged 25–49-years regardless of population type or region [9].

In Uganda, the prevalence of self-reported STIs has remained persistently high, with an increase from 22% in 2006 to 27% in 2011 [10], while up to 1.5 million cases of STIs were reported between 2015 and 2017 [2, 11]. This high prevalence of STIs and associated adverse health outcomes makes STI control a public health priority. Notably, STIs increase the risk of HIV acquisition and are the leading cause of disability-adjusted life years (DALYs) among women of reproductive age [4, 12]. Moreover, STIs acquired from regular partners account for up to 70% of the burden of female infertility [13]. Through several evidence-based strategies, such as ensuring community awareness on risks, prevention strategies, and treatment of STIs, this high burden of STIs and their effects are largely preventable [2, 4, 14].

The major components of Uganda’s current strategy on STI /HIV management include primary prevention strategies such as vaccination for Human papillomavirus and Hepatitis B, male circumcision, and behavioral change communication. Syndromic management, which involves the use of signs and symptoms rather than laboratory tests is also used to guide the treatment of STIs [15]. Despite these interventions, STIs are on an upward trend [8] and although the incidence is highest among young people, they have the least access to quality STI prevention and management services [9]. Management of STIs at public facilities where most youths seek these services is known to be poor, even when health workers are adequately trained and drugs and other consumables are adequately stocked and supplied [16]. The poor services are attributed to multiple barriers including the stigma associated with seeking STI services, confidentiality concerns, and method of specimen collection, conflicting school/work, and clinic schedules as well as the inability to pay for services [3, 17]. Moreover, routine screening for asymptomatic infection is not performed [18] and a large number of people with STIs remain asymptomatic and therefore undiagnosed [12]. These asymptomatic cases are more frequent among youths especially young women compared to older people [19].

Some studies have been conducted on STIs in Uganda, but few of these studies have addressed the burden and their correlates among youths aged 15–24 years. Moreover, there is currently no study that has examined the prevalence of STIs among sexually young people using nationally representative data [20]. To understand the prevalence of STIs and their correlates among youths and inform public health planning and effective interventions, this study examined the trends and correlates of STIs among sexually active female youths aged 15–24 years who participated in 2006, 2011, and 2016 Uganda Demographic and Health Survey (UDHS).

## Methods

### Study design and data sources

This study utilized secondary data from the Uganda Demographic and Health Surveys (UDHS) round of 2006, 2011, and 2016. These are multi-stage nationally representative surveys of households conducted in Uganda every 5 years and collect information on population health along with the socioeconomic and demographic characteristics of respondents. All women aged 15–49 years in sampled households provide self-reported information in the individual woman’s questionnaire about the different STIs they have been experienced within the last 12 months of the survey. The questions focused on whether the women had experienced any genital sores, genital discharge or both or other varginal complaints after sexual intercourse, during the study period. The data sets used did not include information on HIV, hence its exclusion from the self-reported STIs in this study. The analysis in this paper was based on the women’s questionnaire and captured detailed information on respondents, socio-demographic characteristics, sexual behavior, wealth status, maternal and child health as well as different health outcomes including STIs in the last 12 months before each survey [15, 21, 22].

With permission obtained online, the UDHS datasets were downloaded from the DHS website https://www.dhsprogram.com. These surveys were conducted by the Uganda Bureau of Statistics (UBOS) in collaboration with the Ministry of Health (MOH), with technical support and funding from the Government of Uganda, the United States Agency for International Development (USAID), the United Nations Children’s Fund (UNICEF), and the United Nations Population Fund (UNFPA).

### Study population

The study population included sexually active female youths (adolescent girls and young women) aged 15–24 years. We included minors (participants aged between 15 and 17 years) but with consent from their parents or guardians. Respondents above aged 25 and above and those who were not sexually active were excluded.

### Study setting and sample size determination

**Fig. 1:**
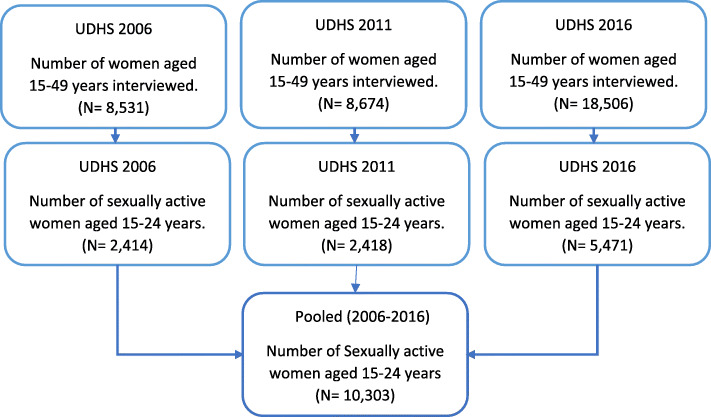
Flow chart for the selection of the study population

This was a nationally representative survey carried out in Uganda. Uganda has a projected population of 41.5 million people according to the 2016 population survey [15]. .For a representative sample, a two-stage cluster sampling method was used, using the Uganda National Population and Housing Census (NPHC) sampling frame, which is a complete list of all census enumeration areas (EAs). In Uganda, EAs is a geographic area covering an average of 10 households. At the time of the recent NPHC, Uganda was divided into 112 districts, which were further divided into 15 sub-regions for this survey. Randomly selected nationally representative samples of households were used in each of the surveys [15, 21, 22]. For analysis in this study, data on sexually active female youths aged 15–24 years for the years 2006, 2011, and 2016 were extracted for analysis. The flow chart for selection of study population is highlighted in Fig. 1.

### Study variables

We extracted data from the individual women’s data set and particularly obtained information for women in the age category 15–24 years at the time of each survey. Our main outcome was the “self-reported prevalence of STIs” within 12 months of the survey. A participant was considered to have had STIs if she reported one or more of the following syndromes: abnormal vaginal discharge, genital ulcer or sores, or any other complaints in the varginal area following sexual contact in the past 12 months before data collection. The definition excludes HIV and bacterial Vaginosis which are difficult to diagnose without laboratory tests. This was used to construct a binary outcome with values of 0 for “no” and 1 for “yes”. The correlates of STIs examined in all the datasets included socio-demographic and sexual behavior characteristics. The sociodemographic factors included age (categorized as 15–19 and 20–24), type of place of residence (categorized as Rural or Urban), region, religion, marital status, and education, type of marriage, household wealth quintile, and spousal age. The level of education was recorded as no education, primary level, or secondary or higher level. Marital status was classified into married/living with a partner, not married or other, and type of marriage as polygamous or not. The regions were coded as Kampala, East, North, West, and Central, while religion as Christians, Muslims, and others, while wealth quintiles were grouped into poor, middle, and rich. Sexual behavior variables included age at first sexual intercourse which was grouped into < 15 years, 15–19 years, and 20–24 years, the number of sexual partners categorized into 1 or more, the total number of lifetime partners classified into 1 and 2 or more, recent sexual activity into “not active in last 4 weeks” or “active in the last 4 weeks”, heard of STIs (yes or no) and condom use which was grouped into “yes” or “no”. consistent condom use was defined as always using a condom at every sexual intercourse with every sexual partner.

### Statistical analysis

All statistical analyses were performed using STATA version 14.0 software. To ensure the representativeness of the population, we used the svyset command to match the multistage cluster sampling design method. In the svyset command, we specified the sample weights (to 6 decimal points) and then used cluster as the primary sampling unit and specified the strata (represented by different regions of Uganda). The different data sets were checked for completeness and consistency, cleaned, coded, and appended for pooled analysis [15, 21, 22]. The unit of analysis was the individual sexually active female youth aged 15-24 years. Descriptive analysis was performed to report frequencies and proportions of STIs for each survey year and generate frequency tabulations to show the trends of STIs and describe the covariate and their distributions over the three survey periods 2006, 2011, and 2016. Bivariate analysis was then conducted for each of the data sets separately using logistic regression to determine the association between the STI status and its correlates. All significant variables with *p*< 0.05 at a 95% confidence interval for either of the data sets were selected for inclusion in the multivariate analysis. In the multivariate analysis, three models were fitted in steps to determine how they independently predicted STIs among the youths using the pooled dataset. Model I contained the year of the interview as the main correlate of STIs to determine its independent effect on the prevalence of self-reported STIs. In Model II, sexual behaviors were added to the first model, and in Model III, sociodemographic characteristics were added to produce the final adjusted model. This model included the correlates, adjusting for sexual behaviors, and sociodemographic characteristics. The pooled data were also used to analyze trends in the prevalence of STIs [23]. Adjusted odds ratios (aOR) and 95% confidence intervals (CI) were reported.

## Results

### Distribution of socio-demographic characteristics

We analyzed data for sexually active young women aged 15–24 years from all three surveys. The total sample size was 10,303 female youths: 2414 in 2016, 2418 in 2011, and 5471 in 2016 (Table 1). More than half (64%) of the respondents were aged 20–24 years and had a mean age of 20.4 (SD=0.03). Over 70% of the women in each survey were from rural residences (80% in 2006, 76.6% in 2011, and 73.6% in 2016). The majority of respondents were Christians 8691(84.4%), were married or living with partner 6328 (61.4%), and employed 7095(68.7%). Regarding education, 3679 (35.7%) had received secondary education or higher (Table 1).

**Table 1:** Distribution of Selected Socio-demographic characteristics; pooled and by year of the survey

Demographic Characteristics	Frequencies (Percentages)			
	2006 (*n* = 2414)	2011 (*n* = 2418)	2016 (*n* = 5471)	Pooled (*N* = 10,303)
**Age group**				
15–19	833(34.5)	923(38.2)	1948(35.6)	3704(36.0)
20–24	1581(65.5)	1495(61.8)	3523(64.4)	6599(64.0)
**Marital status**				
Not married	886(36.7)	918(37.8)	2176(39.8)	3975(38.6)
Married/livingtogether	1528(63.3)	1505(62.2)	3294(60.2)	6328(61.4)
**Residence**				
Urban	482(20.0)	565(23.4)	1444(26.4)	2491(24.2)
Rural	1932(80.0)	1854(76.6)	4026(73.6)	7811(75.8)
**Region**				
Kampala	263(10.9)	291(12.0)	312(5.7)	865(8.4)
Central	467(19.3)	507(21.0)	1354(24.8)	2328(22.6)
East	590(24.4)	650(26.9)	1543 (28)	2783(27.0)
North	477(19.7)	410(17.0)	1025(18.7)	1912(18.6)
West	618(25.6)	560(23.2)	1237(22.6)	2415(23.4)
**Religion**				
Christian	2068(85.7)	2035(84.2)	4587(83.9)	8691(84.4)
Muslim	308(12.8)	360(14.9)	826(15.1)	1494(14.5)
Other religions	36(1.5)	22.6(0.9)	57(1.0)	116(1.1)
**Education**				
No education	229(9.5)	110(4.5)	157(2.9)	496(4.8)
Primary education	1522(63.1)	1457(60.2)	3149(57.6)	6127(59.5)
Secondary education	562(23.3)	741(30.6)	1766(32.3)	3069(29.8)
Higher	101(4.2)	111(4.6)	398(7.3)	610(5.9)
**Literacy**				
Cannot read at all	814(33.7)	684(28.3)	1351(24.7)	2850(27.7)
Able to read	1483(61.5)	1695(70.1)	4091(74.8)	7269(70.6)
Other (blind or visually impaired)	116(4.8)	39(1.6)	27.8(0.5)	183(1.8)
**Wealth index**				
Poor	943(39.1)	863(35.7)	2101(38.4)	3907(37.9)
Middle	398(16.5)	463(19.2)	924(16.9)	1785(17.3)
Rich	1073(44.4)	1092(45.1)	2446(44.7)	4610(44.7)
**Employment**				
No	551(22.9)	921(38.1)	1724(31.5)	3196(31.1)
Yes	1853(77.1)	1496(61.9)	3747(68.5)	7095(68.9)

Source: Uganda Demographic Health and Health surveys (UDHS) 2006, 2011, 2016

### Trends in sexual behaviours and STIs

Half of the respondents in all three separate and pooled datasets (50.1% pooled, and 47.0, 47,8 and 52.5 in 2006, 2011, and 2016, respectively) reported more than one lifetime sexual partner, and only 10.43% used condoms consistently (always) with their most recent partner. Likewise, in the pooled data, more than half (55.6%) of the respondents reported having been sexually active in the last 4 weeks before the interview, consistent with 56.7, 54.5, and 55.6% reported in 2006, 2011, and 2016, respectively. The majority (72.9%) of the sexually active female youths in the pooled dataset had their first sexual intercourse between the ages between 15 and 19. Approximately 71.0%, 68.1, and 74.0 had their first sexual encounters in 2006, 2011, and 2016, respectively (Table 2).

**Table 2:** Trends in sexual behaviors and STIs among Ugandan women age 15–24 years, UDHS 2006–2016

Characteristics	Frequencies (Percentages)			
	2006 (*n* =2414)	2011 (*n*=2418)	2016 (*n* = 5471)	Pooled (*N*=10,303)
**Age at first sex**				
< 15	566(23.5)	512(21.2)	962(17.6)	2040(19.8)
15–19	1713(71.0)	1644(68.1)	4046(74.0)	7402(71.9)
20–24	134(5.6)	259(10.7)	460(8.4)	854(8.3)
**Recent sexual activity**				
Not active	1044(43.3)	1098(45.5)	2431(44.4)	4573(44.4)
Active in last 4 weeks	1366(56.7)	1318(54.5)	3038(55.6)	5722(55.6)
**Number of sex partners**				
Dont have	280(11.6)	319(13.2)	603(11.0)	1203(11.7)
Only one	2070(85.8)	2021(83.7)	4648(85.0)	8739(84.8)
2 or more	63(2.6)	76(3.1)	220(4.0)	358(3.5)
**Total number of Lifetime partners**				
only one	1276(53.0)	1262(52.2)	2598(47.5)	5136(49.9)
2 or more	1134(47.0)	1155(47.8)	2872(52.5)	5161(50.1)
**Number of lifetime marriages/unions**				
Only one	1593(90.0)	1533(90.0)	3470(90.8)	6596(90.4)
2 or more	177(10.0)	171(10.0)	350(9.2)	698(9.6)
**Contraceptive knowledge**				
knows no method	38(1.6)	29(1.2)	20(0.4)	87(0.8)
knows other method	8(0.3)	1(0.0)	3(0.1)	12(0.1)
knows modern method	2368(98.1)	2389(98.8)	5448(99.6)	10,205(99.1)
**Consistent condom use**				
No	2204(91.3)	2158(89.2)	4866(89.0)	9228(89.6)
Yes	210(8.7)	260(10.8)	604(11.0)	1074(10.4)
**Heard about STIs**				
No	11(0.4)	1(0.1)	9(0.2)	21(0.2)
Yes	2403(99.6)	2416(99.9)	5461(99.8)	10,280(99.8)
Do not know		1(0.1)		1(0.0)
**Had genital sore**				
No	2072(86.1)	2034(84.2)	4844(88.6)	8950(87.0)
Yes	336(13.9)	381(15.8)	625(11.4)	1342(13.0)
**Had genital discharge**				
No	2121(88.4)	2079(86.2)	4710(86.2)	8911(86.7)
Yes	279(11.6)	334(13.8)	757(13.8)	1370(13.3)
**Has had any STIs in the last 12 months**				
No	1882(78.0)	1541(63.7)	4205(76.9)	7628(74.0)
Yes	532(22.0)	878(36.3)	1265(23.1)	2675 (26.0)

Source: Uganda Demographic Health and Health surveys (UDHS) 2006, 2011, 2016

### Trends in the weighed prevalence of STIs among female youths aged 15–24 years old

**Fig. 2:**
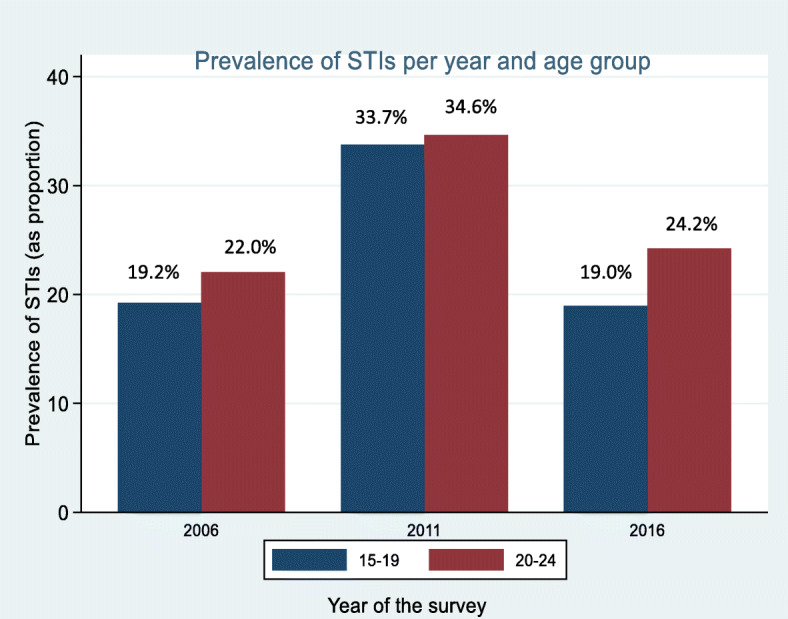
Trends in prevalence of STIs among female youths aged 15–24 years by survey year and age group

The weighted prevalence of the STIs showing their distribution by year, age group, and region and sexual behaviours are reported in Table 3. The table shows a weighted pooled STI prevalence of 26, 95%CI (24.8–27.1). Among the curable STIs assessed, a prevalence of 13.0, 95%CI (12.2–13.9), and 13.3, 95%CI (12.5–14.2) were reported for genital ulcers and genital discharge, respectively. As shown in Table 3 and Fig. 2, about 22.0%, % of youth women reported an STI in 2006. This increased to 36.3% in 2011, before declining to and 23.1% reported a sexually transmitted infection in 2006, 2011, and 2016 respectively. Between 2006 and 2011, there was evidence of change (+ 14.3%, *p* < 0.001) in STI prevalence before a significant reduction (− 12.0%, *p*< 0.001).

**Table 3:** Prevalence of sexually transmitted infections among female youths aged 15–24 years, UDHS 2006–2016

Characteristics	Weighted Prevalences/percent (95% CI)		
	Any STI	Genital sores	Genital Discharge
**Year of interview**			
2006	22.0(19.7–24.6)	13.9(12.1–16.0)	11.6(10.0–13.4)
2011	36.3(33.7–38.9)	15.8(13.8–18.0)	13.8(12.1–15.7)
2016	23.1(21.7–24.7)	11.4(10.4–12.5)	13.8(12.7–15.1)
**Pooled**	**26.0(24.8–27.1)**	**13.0(12.2–13.9)**	**13.3(12.5–14.2)**
**Age group**			
15–19	23.6(21.8–25.4)	10.9(9.7–12.2)	10.8(9.6–12.1)
20–24	27.3(25.9–28.8)	14.2(13.1–15.4)	14.7(13.7–15.9)
**Region**			
Kampala	28.4(24.4–32.7)	15.1(12.0–18.8)	16.8(14.2–19.7)
Central	32.3(29.4–35.3)	16.4(14.4–18.8)	18.3(16.3–20.4)
East	26.8(24.7–29.1)	13.5(12.0–15.1)	11.2(9.9–12.7)
North	16.0(14.2–17.8)	7.2(6.0–8.5)	7.9(6.7–9.4)
West	25.9(23.7–28.3)	13.2(11.4–15.1)	14.0(12.4–15.9)
**Residence**			
Urban	26.8(24.5–29.3)	12.5(10.9–14.4)	16.4(14.4–18.6)
Rural	25.7(24.4–27.0)	13.2(12.2–14.2)	12.3(11.5–13.2)
**Marital status**			
Not married	23.6(21.9–25.4)	11.6(10.3–12.7)	12.6(11.4–13.9)
Married	27.4(26.0–28.9)	14.0(13.0–15.2)	13.8(12.8–14.8)
**Total number of sexual partners**			
Only one	19.5(18.2–20.9)	9.6(8.6–10.^)	9.0(8.2–10.0)
2 or more	32.4(30.7–34.2)	16.5(15.2–17.9)	17.6(16.3–19.0)
**Recent sexual activity**			
Not active	22.2(20.7–23.8)	10.3(9.2–11.4)	10.8(9.7–11.9)
Active in the last 4 weeks	28.9(27.4–30.5)	15.3(14.2–16.5)	15.3(14.2–16.5)
**Religion**			
Christian	25.0(23.8–26.3)	12.5(11.6–13.5)	12.9(12.0–13.7)
Muslim	31.0(27.8–34.4)	15.7(13.7–18.0)	15.5(13.2–18.2)
Other	33.7(24.1–44.9)	17.8(11.2–27.0)	19.8(12.0–30.8)

Source: Uganda Demographic Health and Health surveys (UDHS) 2006,2011,2016

**Fig. 3:**
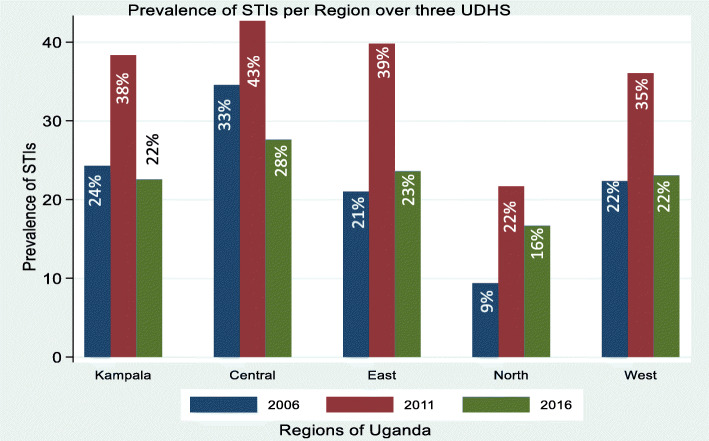
Regional Trends in the Prevalence of STIs among female youths aged 15–24 years by survey year

In general, the prevalence of STIs) among youths 20–24 years (27.3, 95%CI (25.9–28.8) was significantly higher than that for young adolescents, 23.6, 95%CI (21.8–25.4). Additionally, youths in the central region, 32.3, 95%CI (29.4–35.3), and Kampala 28.4, 95%CI (24.4–32.7) reported a higher prevalence of STIs compared to other regions, while the northern region had the lowest prevalence of STIs 16.0, 95%CI (14.2–17.8) (Fig. 3).

### Relationship between STIs and different correlates and among Ugandan female youths

In the bivariate analysis, recent sexual activity in the last 4 weeks prior to the interview, lifetime number of marriages, and the total lifetime number of sex partners were significant in all surveys while age group, region, marital status, religion, education, age at 1st sex, and employment were significant in either one or two of the survey datasets. Condom use was not significant in the bivariate analysis across all datasets (Table 4).

**Table 4:** Bivariate analysis showing Relationship between reporting of STIs and selected correlates

Variables	2006		2011		2016	
	OR (95% CI)	***P***-value	OR (95% CI)	***P***-value	OR (95% CI)	***P***-value
**Age group**						
15–19	1		1		1	
20–24	1.2(1.0–1.5)	0.104	1.0(0.8–1.3)	0.847	1.4(1.2–1.7)	**< 0.001**
**Residence**						
Urban	1		1		1	
Rural	1.1(0.8–1.6)	0.644	1.0(0.7–1.3)	0.822	0.9(0.7–1.1)	0.153
**Region**						
Kampala	1		1		1	
Central	1.6(1.0–2.7)	0.047	1.2(0.8–1.8)	0.347	1.3(0.9–1.8)	0.103
East	0.8(0.5–1.4)	0.457	1.1(0.7–1.5)	0.744	1.1(0.8–1.4)	0.698
North	0.3(0.2–0.6)	**< 0.001**	0.4(0.3–0.7)	**0.001**	0.7(0.5–0.9)	**0.015**
West	0.9(0.5–1.5)	0.680	0.9(0.6–1.3)	0.604	1.0(0.8–1.4)	0.843
**Religion**						
Christian	1		1		1	
Muslim	1.8(1.3–2.5)	**0.001**	1.4(1.1–1.9)	**0.013**	1.2(0.9–1.5)	0.203
Other	2.3(0.9–5.6)	0.069	1.4(0.6–3.2)	0.413	1.3(0.6–2.7)	0.462
**Education**						
No education	1		1		1	
Primary	1.2(0.8–1.8)	0.476	1.6(1.0–2.5)	0.067	1.6(1.0–2.7)	0.051
Secondary	1.1(0.7–1.7)	0.816	1.0(0.6–1.6)	0.994	1.9(1.2–3.2)	**0.010**
Higher	0.9(0.5–1.7)	0.695	1.1(0.5–2.3)	0.802	1.3(0.7–2.2)	0.450
**Employment**						
No	1		1		1	
Yes	0.9(0.7–1.3)	0.730	0.8(0.7–1.1)	0.139	1.2(1.0–1.5)	**0.024**
**Marital Status**						
Not married	1		1		1	
Married/living	1.5(1.2–2.0)	**< 0.001**	1.1(0.9–1.5)	0.336	1.4(1.2–1.7)	**0.001**
**Age at 1st sex**						
< 15	1		1		1	
15–19	0.8(0.6–1.0)	0.106	0.8(0.6–1.1)	0.184	0.8(0.6–0.9)	**0.004**
20–24	0.5(0.3–0.9)	**0.031**	0.8(0.5–1.1)	0.210	0.7(0.5–0.9)	**0.016**
**Recent sexual activity in the last 4 weeks**						
Not active	1		1		1	
Active	0.6(0.5–0.8)	**< 0.001**	0.8(0.6–0.9)	**0.007**	0.7(0.6–0.8)	**< 0.001**
**Lifetime number of marriages/unions**						
Once	1		1		1	
> once	1.6(1.1–2.5)	**0.019**	1.7(1.2–2.5)	**0.005**	1.3(1.0–1.7)	**0.049**
**Total lifetime sexual partners**						
Only one	1		1		1	
2 or more	2.5(2.0–3.2)	**< 0.001**	1.9(1.6–2.4)	**< 0.001**	1.9(1.6–2.2)	**< 0.001**
**Condom use**						
No	1		1		1	
yes	1.0(0.7–1.5)	0.896	0.9(0.6–1.3)	0.605	0.8(0.6–1.0)	0.071

Source: Uganda Demographic Health and Health surveys (UDHS) 2006, 2011, 2016

*ref* Reference

In multivariable analysis, the year of the interview (2011), being sexually active in the last 4 weeks, having 2 or more lifetime sexual partners, being from the northern region of Uganda, and being affiliated with the Muslim faith and other religions were significantly associated with STI status among youths aged 15–24 years. Notably, in the year 2011, participants were more likely to report an STI across all models (OR: 1.9, 95% CI (1.5–2.3)) compared to 2011 and 2016, both had a similar but non-significant odds ratio. Compared with female youths who reported having one lifetime sexual partner, the odds of having an STI was higher among those who reported having 2 or more lifetime partners (OR:1.6, 95%CI (1.4–1.9). Likewise, the odds of reporting an STI were slightly higher among female youths who were sexually active in the last 4 weeks prior to the respective survey (OR:1.3, 95% CI (1.1–1.6)). Besides the sexual behaviours, the youths affiliated with the Muslim faith (OR: 1.3, 95% CI (1.1–1.6)) and other religions (OR: 1.8, 95% CI (1.1–2.9) had higher odds of reporting an STI compared to those affiliated to Christianity. On the other hand, the northern region was found to be inversely associated with reporting any STI among female youths aged 15–24 years with youths from Northern Uganda having 0.5 times lower odds of reporting an STI compared to other regions (OR 0.5, 95%(0.3–0.7). Although statistically significant (*p*< 0.05) in the different survey years in the bivariate analysis, marital status, age at first sex, age group, and having more than one lifetime partner, showed no statistical significance in the adjusted models (Table 5).

**Table 5:** Logistic regression analysis showing the relationship between reporting of STIs and selected correlates of STIs among female youths aged 15–24 years in Uganda using pooled data (Model building)

Selected factors	Model 1		Model 2		Model 3 (fully adjusted model)	
	OR (95% CI)	*P*-value	OR (95% CI)	*P*-value	OR (95% CI)	*P*-value
**Year of interview**						
2006 (ref)	1.0		1.0		1.0	
2011	2.0(1.7–2.4)	**< 0.001**	1.9(1.5–2.3)	**< 0.001**	1.9(1.5–2.3)	**< 0.001**
2016	1.1(0.9–1.3)	0.462	1.0(0.9–1.2)	0.791	1.0(0.8–1.2)	0.836
**Age at first sex**						
< 15 (ref)			1.0		1.0	
15–19			0.9(0.8–1.0)	0.153	1.0(0.8–1.2)	0.739
20–24			0.9(0.7–1.2)	0.585	1.1(0.8–1.4)	0.731
**Recent sexual activity in the last 4 weeks**						
Not active (ref)			1.0		1.0	
Active			1.3(1.1–1.5)	**< 0.001**	1.3(1.1–1.6)	**0.002**
**Lifetime Number of marriages/unions**						
Once (ref)			1.0		1.0	
more than once			1.1(0.9–1.3)	0.348	1.1(0.9–1.3)	0.522
**Total number of life partners**						
Only one (ref)			1.0		1.0	
2 or more			1.9(1.6–2.1)	**< 0.001**	1.6(1.4–1.9)	**< 0.001**
**Residence**						
Urban (ref)					1.0	
Rural					1.0(0.8–1.2)	0.644
**Region**						
Kampala (ref)					1.0	
Central					1.2(0.9–1.7)	0.246
East					0.9(0.6–1.3)	0.615
North					0.5(0.3–0.7)	**< 0.001**
West					1.0(0.7–1.4)	0.791
**Age group**						
15–19 (ref)					1.0	
20–24					1.0(0.8–1.2)	0.846
**Education**						
No education (ref)					1.0	
Primary					1.2(0.9–1.6)	0.168
Secondary					0.9(0.7–1.3)	0.697
Higher					0.8(0.5–1.3)	0.319
**Marital status**						
Not married (ref)					1.0	
Married					0.9(0.7–1.1)	0.175
**Religion**						
Christian (ref)					1.0	
Muslims					1.3(1.1–1.6)	**0.009**
Other					1.8(1.1–2.9)	**0.031**

Source: Uganda Demographic Health and Health surveys (UDHS) 2006, 2011, 2016

*ref* Reference

## Discussion

In this study, we examined the trends in the prevalence and correlates of STIs among female youths aged 15–24 years in Uganda (2006–2016). Findings indicate that the overall prevalence was high (26.0%); highest in 2011 and the lowest in 2006. Having 2 or more life partners, older age, sexual activity in the last 4 weeks, being a Muslim or belonging to other religions other than Christianity, were positively associated with reporting an STI while being from Northern Uganda was negatively associated with reporting any STI among the female youths aged 15–24 years.

In this study, we found that the prevalence of STIs among youths was high, with the highest prevalence found among youths 20–24 years old. This is consistent with several studies [24–28] showing that the prevalence of STIs was higher among young people aged 20–24 years compared to those 15–19 years. Surprisingly, the prevalence of STIs in 2011 was higher than in other years. There was a notable increase in prevalence between 2006 and 2011 and the decline later in 2016. Considering the relationship between HIV and STIs, the high STI prevalence in 2011 may have been due to the higher HIV prevalence around 2011 [29]. It was also confirmed in a study in South Africa that HIV infection is a risk factor for STIs and likewise, STI infection is a risk factor for HIV acquisition [30, 31]. It is also probable that there was laxity in sexual and reproductive health service promotion programs but the decline in 2016 is probably due to increased uptake of sexual and reproductive health (SRH) among young people, leading to improved knowledge of HIV/STI, and promotion of safer sex following the SRH/HIV integration in Uganda in 2012 [32].

Both self-reported and laboratory-diagnosed STIs have been consistently high among young people with a history of having multiple lifetime partners, as seen in evidence from several studies over the years [5, 7, 33, 34]. The results from this study equally reveal that throughout all the survey years and in the pooled dataset, female youths aged 15–24 with a history of 2 or more lifetime sex partners reported higher cases of STIs than those who reported having only one sexual partner. This suggests that those with multiple partners may not be taking strict measures such as consistent condom use to protect themselves from STIs. A study using nested health and the demographic survey also corroborates our findings [24]. Having multiple sexual partners provides an opportunity for transmission of STIs [20, 35]. Improving partner notification supports and practices may therefore be of paramount importance in miinmizing the risk of STIs/HIV.

In Uganda, a study using the 2016 UDHS data revealed that engagement in sexual activities in the last 1 month prior to the survey was significantly associated with self-reported STI status [2]. This corroborates findings from our analysis of the pooled dataset, including data from the 2006, 2011, and 2016 UDHS that show an association between recent sexual activity in the last 4 weeks and self-reported STI status. This is also consistent with a study by Lewis [36] that revealed an association between STIs with recent unprotected sex.

Apart from sexual behaviours, some sociodemographic characteristics including religion and region of residence were associated with STI status among female youths aged 15–24 years.: Findings from this study reveal that women reporting Muslim and other non-Christian religions had higher odds of STIs. This may be attributed to several factors, including the higher observed proportion of Muslim women reporting 2 or more lifetime sexual partners, compared with women who practice Christianity in our study (*p*=0.049) and in previous studies [20]. Moreover, a DHS study in Ethiopia indicated that Muslim affiliation is associated with earlier engagement in sex among adolescents compared to orthodox Christians [37]. Strategies to minimize the risk of STIs may need to be culturally informed and adapted to reach young Muslim women and their partners.

In corroboration with other studies conducted in Uganda [2, 7, 10], our study shows an inversely significant association between STI status and coming from the northern region of Uganda. The northern region is classified among the rural regions of Uganda, so our results are consistent with findings from other studies conducted in Uganda [38, 39], which revealed that living in rural regions, especially northern Uganda, was protective against STI acquisition. Misinde [7] in his study argues that the low prevalence of STIs in northern Uganda may be due to cultural reasons, including strong norms against sexual relationships for young women. On the other hand, youths in urban areas tend to be heterosexually active and this increases their chances of contracting STIs [20]. In Malawi, studies show that females from urban richer wealth quintiles were more likely to report multiple sexual partnerships which were found to be a risk factor for HIV and other STIs [20, 40]. Its documented that some young females in this settings engage in transactional sex as a means of survival against poverty [20]. .This then exposes them to a high risk of STIs/HIV.

### Strengths and limitations of the study

The main strength of this study is that it uses data from the Uganda demographic and health surveys, which are nationally representative studies and have large datasets that increase the power. Data from three different survey years were used for analysis, which allows for a better comparison of prevalence outcome variables and risk factors across the different years. The study however had its limitations; in this study, cross-sectional data were used, which may have introduced recall bias due to participants failing to remember certain exposures, or falsely recalling events influenced by having experienced the outcome and this may result in an underestimation of STIs prevalence. Additionally, the cross-sectional data used only show associations but not causation. Another limitation is that the outcome variable of self-reported STIs among female youths may introduce bias due to under-reporting of STIs hence resulting in low prevalence. Moreover, reporting of symptoms to determine STI status as used in this study is more likely to miss out on asymptomatic STI cases or those cases that would have been identified if participants had been diagnosed by the health worker. Despite these limitations, the results from this data provide insight into the prevalence and correlates of STIs among female youths aged 15–24 years in Uganda across the years. Finally, a complete case analysis of secondary data was used in this study, so we didn’t have information and control over data quality. However, some variables in the data sets were already imputed, and the sample was large enough to provide enough power to answer the research question.

## Conclusion

The prevalence of STIs was high among female youths aged 15–24 years old, with the highest prevalence registered in 2011 necessitating comprehensive STIs screening, surveillance, and treatment programme to minimize STIs burden in the country. The correlates of STIs among young female youths included the total number of lifetime partners, recent sexual activity, and affiliation to Islam while living in the northern region of Uganda were protective against STIs. Given that we found both sociodemographic and sexual behavioral predict STIs, there is a need to adopt a holistic approach towards diagnosis and management of STIs among youths in Uganda. For sexual behaviours, interventions should be targeted towards preventing engagement in multiple sexual relationships. On the other hand, different interventions should also be implemented based on regional STI trends for effectiveness.

## Data Availability

The data used in this study are publicly available with permission from the Demographic and Health Survey Program on https://dhsprogram.com/data/dataset_admin/index.cfm . The authors did not have any special privileges from MEASUREDHS/ICF International and therefore confirm that the data can be accessed by other researchers the same way as the authors did.

## Abbreviations

DHS: Demographic and Health Survey
MOH: Ministry of Health
SRH: Sexual and Reproductive Health
STI: Sexually Transmitted Infection
UNFPA: United Nations Population Fund
UNICEF: United Nations Children’s Fund
